# An overview of resistance to Human epidermal growth factor receptor 2 (Her2) targeted therapies in breast cancer

**DOI:** 10.20517/cdr.2022.09

**Published:** 2022-06-01

**Authors:** Ahmed M. Elshazly, David A. Gewirtz

**Affiliations:** ^1^Department of Pharmacology and Toxicology, Faculty of Pharmacy, Kafrelsheikh University, Kafrelsheikh 33516, Egypt.; ^2^Department of Pharmacology and Toxicology, Virginia Commonwealth University, Richmond, VA 23298, USA.

**Keywords:** HER2+, Targeted therapy resistance, IGF-IR, Src, c-MET, PP2A, CD36, miRNA

## Abstract

Breast cancer (BC) is the second most common cause of cancer-related deaths and the most frequently diagnosed cancer in females. Among breast cancer types, HER2-positive breast cancer occurs in nearly 20% of human breast cancers and is associated with increased aggressiveness, poor prognosis, and shortened overall survival. HER2+ breast cancer is currently managed with multidisciplinary treatment strategies including surgery, radiation, chemotherapy, and targeted therapy. Drug resistance remains a continuing challenge, especially to targeted therapy utilizing monoclonal antibodies and tyrosine kinase inhibitors. This review discusses some of the recent molecular mechanisms that are involved in the development of resistance to Her2-targeted therapies including the PI3K/Akt/mTOR pathway, IGF-IR, Src, c-MET, the PP2A family, CD36, p27^kip1^_, _and miRNAs.

## INTRODUCTION

Breast cancer (BC) is the most frequently diagnosed cancer in females and the second most common cause of cancer-related deaths, accounting for 30% of malignancies in women^[1]^. In the U.S, it is estimated that approximately 40,000 women die from breast cancer each year^[2]^. Moreover, in 2020, 2.3 million women were diagnosed with BC worldwide, with the number of deaths reaching 685,000. Near the end of 2020, 7.8 million women diagnosed with BC in the previous five years were alive, making it the most prevalent cancer globally^[3]^.

Breast cancers prognosis and classification rely not only on tumor morphology but also on the expression levels of three proteins, specifically the estrogen receptor, progesterone receptor, and human epidermal growth factor receptor 2 (HER2). Tumors that do not express any of these proteins are classified as triple-negative breast cancers, a form of the disease that is particularly difficult to treat^[4]^.

HER2 (ERBB2) belongs to the ERBB family, which includes epidermal growth factor receptor 1 (EGFR) **(**ERBB1/HER1), HER3 (ERBB3), and HER4 (ERBB4)^[5]^. HER2 shares structural and sequence similarities with the other family members consisting of three regions: an extracellular N-terminal domain, a single transmembrane α-helix domain, and a tyrosine kinase intracellular domain^[6,7]^.

Extracellular ligands have a conserved EGF motif bind with ERBB receptors, causing homo- and heterodimeric interactions between the ERBB receptors in various combinations^[7]^. HER2 has lacks a ligand binding domain and does not require a ligand for activation and may be found in an activated state via homo-dimerization or hetero-dimerization with other members of the ERBB family. Homo- or hetero-dimerization causes autophosphorylation of the tyrosine kinase domains, resulting in the subsequent activation of different signaling pathways, primarily the phosphoinositide 3-kinase/protein kinase B/mammalian target of rapamycin (PI3K/Akt/mTOR) and Ras/Raf/mitogen-activated protein kinase (MAPK) pathways, promoting cell survival, proliferation, differentiation, angiogenesis, and invasion^[8-11]^. Although ERBB receptors are vital regulators for different cellular processes, their dysregulation, as a result of mutations, could lead to the development of cancers^[7]^.

Amplification or overexpression of the human epidermal growth factor receptor-2 occurs in nearly 20% of human breast cancers and is associated with increased aggressiveness, poor prognosis, and short overall survival^[11]^. 

HER2+ breast cancer is currently managed with multidisciplinary treatments that include surgery, radiation, chemotherapy, and targeted therapy^[12]^. Targeted therapy includes monoclonal antibodies such as trastuzumab and tyrosine kinase inhibitors. Although these drugs markedly improve the prognosis of HER2+ breast cancer patients^[13,14]^, a substantial fraction of these patients still suffer from relapse due to intrinsic or acquired resistance to the treatment, particularly in the case of trastuzumab. The majority of patients who achieve an initial response to trastuzumab-based therapy develop resistance within one year^[15-17]^. In fact, 70% of patients with HER2+ BC show intrinsic or acquired resistance to trastuzumab^[18]^.

In this review, we will shed light on some of the molecular mechanisms that are involved in resistance to anti-HER2 therapies, which significantly hinder their efficacy.

## SPECIFIC MECHANISMS OF RESISTANCE

### Dysregulation of the PI3K/Akt/mTOR pathway 

Targeted therapy resistance may occur as a result of the sustained activation of signaling pathways such as the PI3K/Akt/mTOR pathway, despite HER2 blockage, priming a drug escape mechanism^[19-21]^. 

**Figure 1:**
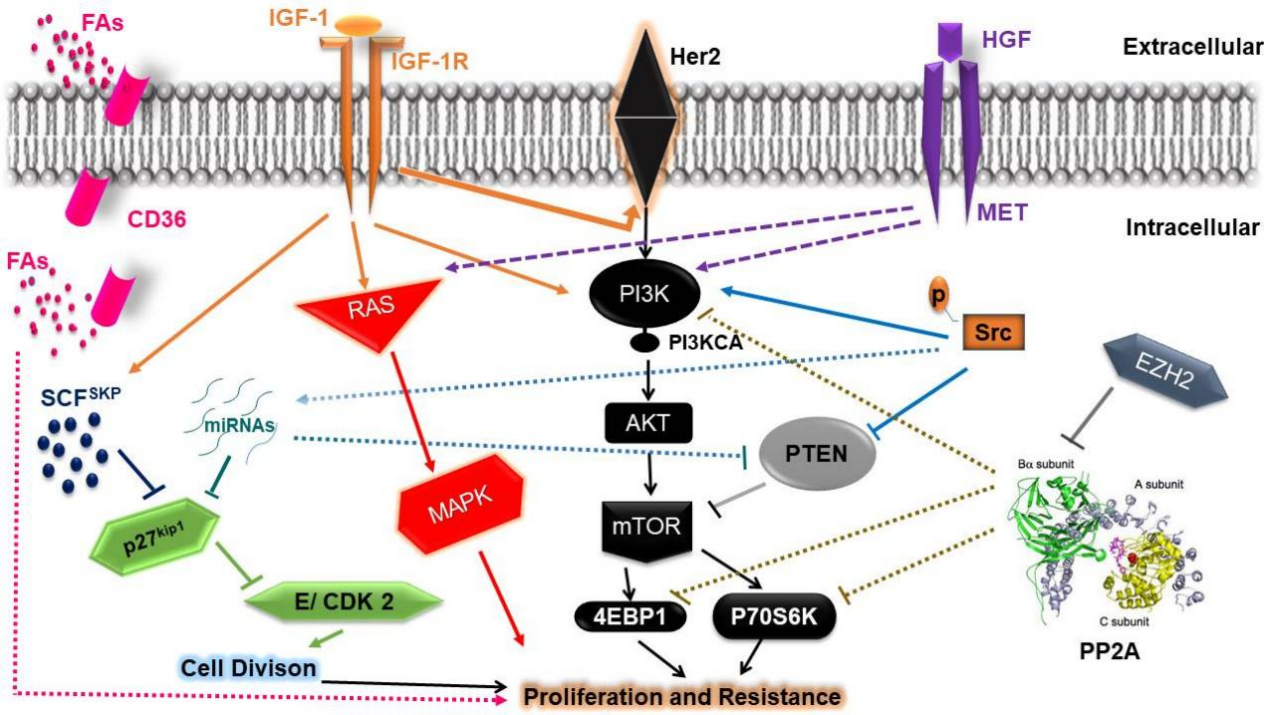
Signaling pathways involved in the development of resistance to Human epidermal growth factor receptor 2 (Her2)-targeted therapy. A central element of resistance appears to be PI3K/AKT/mTOR signaling, which may demonstrate persistent activation through c-MET, IGF-1, p-Src, or interference with PTEN and PP2A mediated suppression of mTOR and downstream signaling at the level of p70S6K and 4EBP1. PP2A activity could also be inhibited by EZH2-mediated slicing of the PP2A regulatory B-subunit. miRNAs and p-Src can also promote the loss of PTEN activity. Resistance could also be mediated through c-MET or IGF1 activation of the RAS/MAPK signaling pathway well as IGF-1; IGF1 can also induce Her2 receptor phosphorylation. p27^Kip1^ expression is reduced via SCF^SKP^ E3 ubiquitin-mediated degradation, which can be augmented by IGF-1 or via miRNAs which are overexpressed through p-Src, causing loss of cyclin E/CDK2 control and promoting cell cycle progression. CD36 contributed to tumor growth and resistance to Her 2 targeted therapies by providing FAs as a critical energy source for tumorigenesis. PTEN: Phosphatase and tensin homolog; IGF: insulin-like growth factor; MAPK: mitogen-activated protein kinase; FAs: fatty acids.

PI3K/Akt signaling dysregulation results in mTOR pathway upregulation and increased mRNA translation leading to enhanced cellular proliferation^[22,23]^, which is mediated by the overexpression of growth factor receptors and loss of the phosphatase and tensin homolog (PTEN) [Figure 1]^[24]^. Breast cancer models of hyperactive PI3K/Akt/mTOR pathway have shown resistance to targeted therapy^[25]^.

The persistent activation of this pathway may result from mutations in genes such as PIK3CA, AKT1, AKT2 amplification, and PTEN loss^[26]^. PTEN loss or PIK3CA mutations are common oncogenic events in HER2+ breast cancer, occurring in approximately 19% and 42% of patients, respectively^[27]^.

PTEN, an mTOR negative regulator, is a tumor suppressor gene whose suppression leds to trastuzumab resistance and shorter survival^[20,28]^. Nagata *et al.*^[29]^ provided compelling evidence supporting the role of PTEN loss in trastuzumab resistance. They showed that PI3K/Akt signaling increased via PTEN downregulation [Figure 1], which resulted in blockage of the growth arrest mediated by trastuzumab. Furthermore, they demonstrated that the absence of PTEN expression was associated with a significantly poorer response to trastuzumab-based therapy in HER2+ BC patients than in those with normal PTEN expression. Moreover, in PTEN-deficient cells, PI3K inhibitors decrease trastuzumab resistance both *in vitro* and *in vivo*^[29]^.

PIK3CA is a gene that encodes the PI3K catalytic subunit. PIK3CA mutations acquired during disease progression are suggested to reflect increased activation of the PI3K pathway [Figure 1]^[19]^. *In vitro* data show that PI3KCA gene mutations and HER2 gene amplification are accompanied by resistance to HER2-targeted therapy^[21,30,31]^. In addition, biomarker analysis from the CLEOPATRA trial showed that PIK3CA mutations were associated with worse survival outcomes in patients with advanced HER2+ breast cancer^[32]^. Moreover, in the EMILIA trial, PTEN loss or PIK3CA mutations were associated with shorter survival and lower overall response rates in patients receiving capecitabine and lapatinib^[33]^.

These data support a role of the dysregulated PI3K/Akt/mTOR signaling pathway in the development of resistance to HER2 targeted agents.

### c-MET 

c-MET (mesenchymal-epithelial transition factor) is a tyrosine kinase receptor encoded by the proto-oncogene MET. Along with RON, c-MET belongs to the MET family, which is widely expressed in epithelial and endothelial cells^[34-36]^. c-MET controls a number of different cellular processes, including replication, survival, and motility^[37]^.

c-MET becomes activated upon binding with its ligand, the hepatocyte growth factor (HGF), triggering a variety of downstream signaling pathways, including PI3K/AKT, Ras/MAPK [Figure 1], Src, signal transducer, and transcription activator^[38-41]^.

Aberrant c-MET activation can contribute to both tumor growth and metastasis^[37]^. For example, c-MET was reported to be highly expressed in HER2+ BC cell lines and in 25% of HER2+ BC patients’ tissues^[42,43]^. Poorly differentiated and invasive cell lines also showed an elevated level of c-MET^[44]^. Clinically, a number of trials demonstrated that c-MET hyperactivity in breast tumors is associated with a lower survival rate^[43,45]^. 

Several experimental findings suggest a role for c-MET in targeted-therapy resistance. Engelman *et al.* showed that c-MET amplification causes HER3-mediated activation of PI3K, and results in gefitinib resistance in lung cancer^[46]^. In addition, c-MET hyperactivity has been reported as a potential contributor to trastuzumab resistance that may be mediated through sustained Akt activation [Figure 1]^[42,43]^. Additionally, c-MET/HGF axis amplification was reported in a cohort of HER2+ BC patients who failed to respond to trastuzumab-based therapies^[42]^. 

Upon treatment with trastuzumab, HER2-overexpressing BC cells may upregulate c-MET, which then protects cells against trastuzumab^[42]^. Moreover, loss of c-MET function is reported to improve the response of these cell lines to trastuzumab^[47]^. 

In studies to demonstrate the significance of c-Met inhibition, Yue *et al.*^[48]^ reported that miR-182 directly targets the c-MET gene in BC cells and that miR-182 downregulation is associated with trastuzumab resistance in BC cells. They utilized miR-182 to reverse the trastuzumab resistance of BC cells in part via targeting c-MET and its downstream PI3K/AKT/mTOR pathway. Their studies showed that c-MET downregulation restored sensitivity to trastuzumab *in vitro* using SKBR3 and BT474 BC cell lines, as well as in xenografted models^[48]^.

Cell lines that have upregulated the c-MET/HGF axis have also demonstrated reduced lapatinib sensitivity, indicating that c-MET activation may decrease the effectiveness of the EGFR/HER2 inhibitors. Conversely, lapatinib or erlotinib combined with foretinib, a c-MET inhibitor, suppressed the growth of these cell lines^[49,50]^.

Many selective c-MET inhibitors are currently under clinical development. Cabozantinib, for example, an inhibitor of c-MET and VEGFR2, is being evaluated, in combination with trastuzumab, in HER2 positive BC patients who suffer from brain metastasis^[50] ^[Table 1]**.**

**Table 1:** A summary of different targets that promote the development of Human epidermal growth factor receptor 2 (Her2)-targeted therapy resistance and drugs to potentially overcome resistance

**Factors**	**Mechanism**	**Drugs**	**Ref.**
PTEN loss	PTEN loss causes loss of PI3K/Akt/mTOR pathway control and its sustained activation	BEZ235	^[29,127]^
PIK3CA mutations	PIK3CA mutations increase activation of the PI3K/Akt/mTOR pathway	BEZ235	^[19,127]^
c-MET	Upon activation with its ligand HGF, c-MET triggers a variety of downstream signaling pathways, including PI3K/AKT, Ras/MAPK, and Src	Cabozantinib	^[38-41,50]^
IGF-IR	Upon ligand binding, IGF-IR initiates signaling through the Ras/MAPK and PI3K/ AKT pathways. IGF1 signaling also elevates the expression of the p27^Kip1^ ubiquitin ligase, SKP2, resulting in the degradation of p27^Kip1^ IGF1 signaling induces the phosphorylation of the HER2 receptor	BMS-754807	^[52,53,54,127]^
p27^kip1^	Alteration in its cellular localization or reduced expression is permissive for the activation of cyclin E/ CDK 2, causing cell cycle progression	MG132	^[63,64,65,56]^
Src	pSrc promotes activation of the PI3K/Akt/mTOR pathway, EGFR, HER2, and HER3 receptors. Src has been found to inhibit PTEN activity and interfere with its membrane localization	Dasatinib Saracatinib	^[29,78,80,81,82]^
PPP2R2B	PPP2R2B downregulation or its silencing by EZH2 causes persistent PI3K/Akt/mTOR pathway activation	EPZ-6438	^[94,95,96-101,102]^
CD36	The CD36-mediated pathway is activated and becomes the major source of FAs uptake rather than FASN-mediated FAs de novo biosynthesis, and provides the cell with needed energy sources, promoting tumor growth and survival	--------	^[111]^
MicroRNAs	MicroRNAs upregulation targets cell cycle regulators such as p57 and p27. miR-221 directly inhibits PTEN	---------	^[69-71,72,118]^

PTEN: phosphatase and tensin homolog; MAPK: mitogen-activated protein kinase; PIK3CA: PIK3 catalytic subunit; HGF: hepatocyte growth factor; IGF-IR: insulin-like growth factor-I receptor; PPP2R2B: regulatory B-subunit of PP2A; pSrc: phosphorylated Src; FAs: fatty acids.

### Insulin-like growth factor 1 receptor signaling

Another pathway involved in targeted therapy resistance is mediated via the Insulin-like growth factor-I receptor (IGF-IR)^[51]^. IGF-IR is a tyrosine kinase receptor that plays a critical role in tumor progression. Upon ligand binding, IGF-IR initiates signaling through the Ras/MAPK and PI3K/ AKT pathways [Figure 1], resulting in cell proliferation and the inhibition of apoptosis^[52]^.

A number of studies support a role of IGF-IR in the development of HER2+ breast cancer resistance. One study demonstrated that IGF1R interacts with and induces phosphorylation of HER2 in trastuzumab-resistant cells [Figure 1], but not in the sensitive parental cells. Moreover, the resistant cells showed more-rapid IGF1 stimulation of PI3K/Akt and Ras/MAPK pathways compared with the parental cells^[53]^. Furthermore, in the *in vitro* model of resistance, IGF1R signaling inhibition either by IGF1R tyrosine kinase suppression or antibody blockade restored sensitivity to trastuzumab^[53]^.

In a study by Lu *et al.*^[51]^, the link between trastuzumab resistance and IGF1R signaling was confirmed. These investigators demonstrated that the growth inhibition mediated by trastuzumab was lost in breast cancer cells having high HER2 and IGF1R levels. Moreover, growth arrest was restored when IGF1- mediated activation of IGF1R was inhibited by IGF-binding protein 3 (IGFBP3)^[51]^. IGF1 signaling was also demonstrated to elevate the expression of the p27^Kip1^ ubiquitin ligase, SKP2, resulting in degradation of the cyclin-dependent kinase inhibitor p27^Kip1^ and loss of growth arrest [Figure 1]^[54]^. p27^Kip1^ belongs to the family of cyclin-dependent kinase inhibitors (CDKIs), which have inhibitory activity towards different CDKs and may function as a tumor suppressor gene by inducing cell cycle arrest^[55]^.

In another study, cyclin-dependent kinase 2 activity was found to be increased in trastuzumab-resistant HER2+ BC cells, accompanied by a reduction in p27^Kip1 ^levels. The addition of exogenous p27^Kip1^ increased sensitivity to trastuzumab. Thus, these data suggest that p27^Kip1^ reduction is associated with trastuzumab resistance, which may be mediated by HER2 and IGF-IR heterodimerization^[56,57]^.

### p27^kip1^

p27^kip1 ^(p27) is a member of the CDKI family^[58]^ that functions as an inhibitor of cell cycle transition from the G1 to S phase by suppressing cyclin E/CDK 2 [Figure 1]^[59]^. The p27^kip1 ^expression level serves as a prognostic marker for BC patients^[60]^. p27^kip1 ^level can be decreased at post-transcriptional levels during G1/S phase progression via ubiquitination and proteasomal degradation after its phosphorylation^[61]^.

Several studies have investigated the role of p27 in targeted-therapy resistance, particularly to trastuzumab and lapatinib in Her2-positive breast cancer.

Trastuzumab-mediated growth arrest appears to depend, in large part, on p27 activity. Trastuzumab causes G1 cell-cycle arrest through increasing the formation of p27-CDK2 complexes^[62]^. Moreover, trastuzumab increases p27 half-life via decreasing its phosphorylation by cyclin E/CDK2, as well as by suppressing the subsequent ubiquitin-mediated degradation^[63]^. 

Yakes *et al*. and Le *et al. *demonstrated that the reduction of p27 levels in SKBR3 HER2+ BC cells by antisense oligonucleotides^[64]^ or by small interfering RNA^[63]^ blocked the growth arrest mediated by trastuzumab. Nahta *et al*. also reported that the trastuzumab-resistant SKBR3 cells showed reduced p27 levels and increased CDK2 activity^[56]^.

Furthermore, Nahta *et al. *showed that transfection-mediated expression of p27 in the resistant cells increased trastuzumab sensitivity^[56,57]^. This is consistent with the findings of Kute *et al.*^[65]^ in which p27 induction by MG132 (a proteasome inhibitor) [Table 1] restored trastuzumab sensitivity, suggesting that downregulation of p27 is likely to result from increased protein degradation. They also reported that the p27 cellular localization might be important for the response to trastuzumab, as trastuzumab-resistant BT474 HER2+ BC cells showed a loss in the nuclear expression of p27^[65]^.

These data are consistent with earlier results in which trastuzumab exposure caused an elevation in p27 levels and nuclear localization in the sensitive cells^[64]^.

In addition, studies by Kute *et al.*^[65]^, Shattuck *et al.*^[42]^, and Lu *et al.*^[51,54] ^suggested that the IGF1R and MET signaling could contribute to p27 downregulation and the development of trastuzumab-resistance, indicating that p27 appears to be a common endpoint for various resistance pathways ^[42,51,53,54]^.

Several studies also showed that Src overexpression activated the proteolysis of p27, which may confer lapatinib-resistance in Her2 + breast cancer^[66]^. It was reported that Src phosphorylates p27 at Tyr74 and Tyr88 to reduce its stability and reduce p27-cyclin E-CDK2 complex formation during G1 phase^[67]^. Moreover, p27 phosphorylation by Src further promotes the phosphorylation of p27 by cyclin E/ CDK 2, resulting in p27 degradation by SCF^SKP^ E3 ubiquitin^[68]^.

miR-221 upregulation has been reported in different tumors and may be involved in tumor progression by affecting expression levels of the cell cycle regulators such as p57 and p27 [Figure 1]^[69-71]^. Interestingly, Huynh *et al.*^[72] ^reported that Src-activated NF-κB may result in miR-221 upregulation in lapatinib-resistant cells [Figure 1]. In addition, the findings of Huynh *et al.* indicated miR-221 involvement, and not the ubiquitination-proteasomal degradation pathway, in p27 downregulation in the lapatinib - resistant cells^[72]^.

These data demonstrated the crosstalk of p27 with the different signaling pathways as well as its role in the development of targeted therapy resistance. 

### Src

The cellular proto-oncogene Src is a non-receptor tyrosine kinase that regulates varied biological processes such as cellular replication, differentiation, and survival^[73,74]^. Aberrant Src activation is considered to be a marked oncogenic event^[75]^. Src is normally found inactivated by the intramolecular binding of its phosphotyrosine (Tyr530) with the Src homology 2 domain^[73]^. The involvement of receptor tyrosine kinases (RTKs) with growth factors such as EGF and PDGF causes Y530 dephosphorylation and consequent Src activation^[76,77]^. The activated Src then autophosphorylates tyrosine 416 residue (Tyr416) in its kinase domain, enabling it to interact with a variety of targets^[73]^. 

In terms of the relationship between Src activation and the response to targeted therapy, one study which involved 57 BC patients showed that tumors with a high level of phosphorylated Src (pSrc) had a poor clinical response and more aggressive disease after trastuzumab therapy. As might have been anticipated, the overall survival was significantly lower than for patients with pSrc-low tumors^[78]^.

Peiró *et al.*^[79]^ demonstrated that Src activation resulted in trastuzumab resistance and poor prognosis in HER2+ breast cancer patients. Moreover, the *in vitro* inhibition of Src restored trastuzumab sensitivity in the resistant cells, with the ability to suppress tumor growth in several preclinical models of resistance. These data are consistent with findings by Zhang *et al.*^[78]^, where expression of the activated SRC in a trastuzumab-sensitive BC cell line, BT474, caused trastuzumab resistance both *in vitro *and in xenografted mouse models. Conversely, sensitivity was restored by Src inhibition^[78]^. In a related finding, in a phase 2 trial of 23 patients with metastatic HER2+ BC, the combination of paclitaxel, trastuzumab, and dasatinib (Src inhibitor) showed high efficacy and success rate^[80] ^[Table 1].

Lapatinib resistance has also been reported in cell lines showing a high level of the activated Src. Lapatinib combined with Saracatinib (Src inhibitor) significantly prolonged the xenografted-mice survival^[81] ^[Table 1].

In efforts to understand the mechanistic relationships between the HER family, IGF-1R, PTEN, and Src, the work of Zhang *et al.*^[78] ^showed that overexpression of EGFR or IGF-1R or PTEN loss caused Src hyperactivation that is monitored by Tyr416 phosphorylation. The activated Src then promoted trastuzumab resistance in BC cell lines via a PI3K/Akt-dependent [Figure 1] or via independent manner^[78]^. These cell lines also showed sensitivity to Src inhibitors^[78]^. Moreover, Src inhibition led to a reduction in EGFR, HER2, and HER3 activation, suggesting that the Src hyperactivity in trastuzumab-resistant cells induces a feedback loop where the active Src causes receptor activation, which, then activate Src.

Studies by Nagata *et al.*^[29]^ and Lu *et al.*^[82]^ also suggest a linkage between Src and PTEN, wherein PTEN can use its protein phosphatase activity to regulate Src phosphorylation. Src has been found to inhibit PTEN activity through tyrosine phosphorylation as well as by blocking the membrane localization of PTEN^[29,82] ^[Figure 1], indicating that Src and PTEN may regulate each other to produce trastuzumab resistance.

These results suggest that Src activation is a common event during the development of targeted therapy resistance and that Src inhibition may be a novel therapeutic strategy.

### The Protein Phosphatase 2A (PP2A) Family

PP2A is a serine/threonine phosphatase family that regulates a variety of cellular processes including growth, metabolism, and apoptosis^[83]^. PP2A family members are also well known for their role as tumor suppressors^[84,85]^, suppressing several oncogenic pathways in carcinogenesis including Wnt, Myc, and PI3K/AKT/mTOR^[86-88]^. 

Structurally, the PP2A family is found as a heterotrimeric complex that consists of scaffolding A-subunit (PPP2R1A), regulatory B-subunit (PPP2R2B), and catalytic C-subunit (PPP2R2C)^[89]^. The PPP2R1A subunit often carries inactivating mutations^[90,91]^, while the PPP2R2C and PPP2R2B subunits may be subjected to epigenetic repression or deletions^[88,92,93]^. These mutations suppress the PP2A tumor suppressor activity, leading to cancer development.

One critical pathway that PP2A controls is the PI3K/AKT/mTOR pathway. The ribosomal protein S6 kinase beta-1 (p70S6K) and eukaryotic translation initiation factor 4E binding protein 1 (4EBP1) regulate two downstream pathways of AKT and mTOR that are vital for cellular proliferation and tumorigenesis^[94,95]^. PP2A can directly dephosphorylate p70S6K and 4EBP1 to maintain the PI3K/AKT/mTOR pathway equilibrium [Figure 1]^[96-101]^. Consequently, PP2A may be linked to targeted-therapy sensitivity in cancer, including monoclonal antibodies and tyrosine kinase inhibitors^[88,90]^. 

In a study by Tan* et al.*^[88]^, the silencing of PPP2R2B by DNA hypermethylation was found to be accompanied by mTOR inhibitor resistance, while Bao *et al.*^[102]^ demonstrated that PPP2R2B downregulation is associated with poor prognosis in HER2+ BC and resistance to HER2-targeted therapy, including both lapatinib and trastuzumab. Additionally, these authors suggest that the persistent activation of both p70S6K and 4EBP1 following HER2-targeted therapy in low PPP2R2B-expressing tumor cells might result in therapy failure.

EZH2 is the catalytic subunit of the polycomb repressive complex 2, which suppresses gene expression via mediating the lysine 27 methylation of histone 3^[103]^. In this study by Tan *et al.*^[103]^, PPP2R2B silencing mediated by EZH2 was shown to be required for both drug tolerance and HER2-targeted therapy resistance [Figure 1]. Moreover, HER2-targeted therapies combined with EZH2 inhibitors in anti-HER2 resistant cell lines and mouse models engrafted with trastuzumab-resistant cells showed significant tumor growth arrest in both cases. These studies suggest that the combination of EPZ-6438 (EZH2 inhibitor) with HER2-targeted therapy might prevent tumor recurrence and metastasis^[102]^.

Taken together, these data indicate the involvement of the PP2A family in the development of HER2-targeted therapy resistance with the potential use of the PPP2R2B expression levels as a predictive marker for the response.

### CD36

Fatty acids (FAs) play a vital role in various biological processes, including cellular signaling, membrane phospholipids synthesis, and energy production^[104,105]^. Generally, two FAs sources are available for cells to meet their energy requirements: exogenously via specialized transporters and/or endogenously via FA synthase (FASN)-mediated de novo biosynthesis^[106]^. In contrast to normal cells (except liver and adipose tissues), which preferentially acquire FAs from exogenous sources, more than 90% of FAs in cancer cells are normally derived from de novo FAs synthesis^[105]^. Tumor cells depend on these two sources not only to sustain their proliferative capacity but also to secure a critical energy source under stress conditions^[107]^.

In terms of exogenous FAs uptake, specific transporters are needed to facilitate FAs movement across the plasma membrane. The most commonly identified transporter is CD36^[108]^, an 88-kDa glycosylated membrane protein that is well-known as a member of the type B scavenger receptor family. CD36 binds several ligands including anionic phospholipids, thrombospondin, and fatty acids^[109]^. 

Vazquez-Martin *et al.*^[110]^ reported that the FASN-mediated FAs de novo biosynthesis is inhibited in response to HER2 inhibition and cancer cells undergo apoptosis, indicating crosstalk between FASN and HER2. However, a recent study found that lapatinib-resistant cells are unresponsive to (-)-C75, which is well-established to suppress FASN^[111]^. Furthermore, the lapatinib-resistant cells showed a significantly higher level of CD36 with an enhanced rate of FAs uptake, as well as an increase in the presence of lipid droplets compared to their sensitive counterparts^[112]^. CD36 expression has also been reported to be increased after anti-HER2 therapy, including lapatinib and trastuzumab, which significantly correlates with a poor prognosis in HER2+ BC patients^[111]^. These data suggest that there is a shift in the metabolic dependence toward CD36-mediated FAs uptake in these cells for maintaining the cellular FAs requirements^[111]^.

CD36 knockdown via siRNA or the small molecule inhibitor, sulfosuccinimidyl oleate (SSO), re-sensitizes lapatinib-resistant cells and induces apoptotic cell death^[111]^. The role of CD36 in lapatinib resistance was also supported by a tumor xenograft study in mice, in which an anti-CD36 antibody markedly sensitized the resistant tumors to lapatinib. Moreover, CD36 knockdown repressed the tumor growth and extended the survival in a HER2+ BC model^[111]^. 

Illustrating the mechanistic aspects of how FAs source alteration in response to targeted therapy ultimately leads to resistance, Feng * et al.*^[111] ^suggested that HER2 activates FASN via phosphorylation, as well as via transcriptional induction. Therefore, HER2 inhibition mediated by trastuzumab or lapatinib causes the suppression of FASN activity. The CD36-mediated pathway is then activated to become the major source of FAs uptake, promoting tumor growth and survival [Figure 1]^[111]^. 

These reports indicated the possible role of CD36 in resistance to anti-Her2 targeted therapy. 

### MicroRNAs 

MicroRNAs (miRNAs) are a class of 22-25 nucleotide RNA molecules that downregulate gene expression^[113]^. miRNAs also play a vital role in the post-transcriptional regulation of mRNA stability and translation efficiency through base-pairing with the complementary locations in the 3’untranslated region of the mRNA^[113,114]^.

miRNAs have been shown to be significantly deregulated in many types of cancer, especially breast cancer, in which deregulated miRNAs are associated with breast cancer metastasis and poor prognosis in some cases, highlighting their critical roles in carcinogenesis, tumor growth, and metastasis^[114-116]^. 

The oncogenic miRNA, miR-221, is one of the few miRNAs that are persistently elevated in malignancies of different tissue including breast cancer^[117]^, and has been suggested to accelerate cancer progression by targeting cell cycle regulators such as p57 and p27 [Figure 1]^[69-71]^. 

Several studies suggest the role of miRNA in the development of targeted therapy resistance. Xingming *et al.*^[118] ^reported that miR-221 is upregulated in breast cancer cells resistant to trastuzumab. In addition, miR-221 directly inhibits PTEN [Figure 1], causing an elevation in motility and invasiveness of HER2+ BC cells. Moreover, miR-221 suppression or the restoration of PTEN expression reversed the malignant phenotypes of HER2+ BC, indicating the critical role of miRNAs in regulating the progression of HER2+ breast cancer^[118]^.

Huynh *et al.*^[72] ^discovered that miR-221 upregulation by the Src/NF-κB pathway contributed to the development of lapatinib resistance by targeting p27 [Figure 1]. Furthermore, miR-221 inhibition by Src inhibitors may serve as a novel therapeutic strategy to overcome targeted-therapy resistance^[72]^. Interestingly, miR-16 has been reported to be involved in regulation of the NF-κB pathway^[119]^. In addition, miR-630 and miR-16 have been shown to play a role in HER2+ BC sensitivity to lapatinib^[120-121]^. The interplay among miR-16, miR-630, and miR-221 in regulating the cellular response to targeted-therapy needs to be further investigated.

## CONCLUSIONS

Her2-targeted therapies are safe and effective drugs for Her2-positive breast cancer; however, de novo or acquired resistance to these agents has limited their clinical efficacy, which ultimately leads to disease relapse and tumor progression. Many molecular mechanisms are incorporated in targeted-therapy resistance including signaling through alternative RTKs^[122,123]^, altered immune response^[122]^, altered antibody-receptor interaction^[123]^, Her2 TK domain mutations^[124]^, HER3 or HER4 overexpression^[125,126]^ and p95HER2 overexpression^[50]^. In this review, we have focused on recent mechanisms that play a role in resistance development and the crosstalk between different signaling pathways that contribute to disease progression and resistance. Current and future strategies for overcoming resistance include switching or combining different Her2-targeted agents and the development of small molecule inhibitors such as BEZ235 (Dual PI3K/mTOR inhibitor)^[127] ^[Table 1], BMS-754807 (IGF1R inhibitor)^[127] ^[Table 1], and EPZ-6438 (EZH2 inhibitor)^[102] ^[Table 1]. These strategies show promise towards improving survival in Her2 positive BC patients.

## DECLARATIONS

### Copyright

© The Author(s) 2022.
